# Miyoshi myopathy associated with spine rigidity and multiple contractures: a case report

**DOI:** 10.1186/s12891-024-07270-y

**Published:** 2024-02-16

**Authors:** Sergey N. Bardakov, Angelina A. Titova, Sergey S. Nikitin, Valentin Nikitins, Margarita O. Sokolova, Vadim A. Tsargush, Elena A. Yuhno, Oleg V. Vetrovoj, Pierre G. Carlier, Yana V. Sofronova, Аrtur А. Isaev, Roman V. Deev

**Affiliations:** 1 Department of Neurology, S.M. Kirov Military Medical Academy, 6 Lebedeva str., St. Petersburg, 194044 Russia; 2 Kazan (Volga Region) Federal University, 18 Kremlyevskaya str., Kazan, 420008 Russia; 3 Research Centre for Medical Genetics, 1 Moskvorechye str., Moscow, 115522 Russia; 4 Avtsyn Research Institute of Human Morphology of Federal State Budgetary Scientific Institution “Petrovsky National Research Centre of Surgery”, 3 Tsyurupy str., Moscow, 117418 Russia; 5 Pavlov Institute of Physiology, Russian Academy of Sciences, 6 Makarova emb, St. Petersburg, 199034 Russia; 6 Neuromuscular Disease Reference Center, University of Liege, and Department of Neurology, St Luc University Hospital, Avenue Hippocrate 10, Brussels, 1200 Belgium; 7 Genetico, 3, Gubkina str., Bldg. 1, Moscow, 119333 Russia; 8 Artgen Biotech PJSC, 3 Gubkina str., Moscow, 119333 Russia; 9 FSBI All-Russian Center for Emergency and Radiation Medicine named after A.M. Nikiforov EMERCOM of Russia, 4/2 Lebedev str., St. Petersburg, 194044 Russia; 10 North-Western State Medical University named after I.I. Mechnikov, 47 Piskarevskij prospect, St. Petersburg, 191015 Russia

**Keywords:** Contractures, *DYSF*, Dysferlinopathy, Limb-girdle muscle dystrophy R2, Spine rigidity

## Abstract

**Background:**

Dysferlinopathy is a phenotypically heterogeneous group of hereditary diseases caused by mutations in the *DYSF* gene. Early contractures are considered rare, and rigid spine syndrome in dysferlinopathy has been previously reported only once.

**Case presentation:**

We describe a 23-year-old patient with Miyoshi myopathy with a rigid spine and multiple contractures, a rare phenotypic variant. The disease first manifested when the patient was 13 years old, with fatigue of the gastrocnemius muscles and the development of pronounced contractures of the Achilles tendons, flexors of the fingers, and extensors of the toes, followed by the involvement of large joints and the spine. Magnetic resonance imaging revealed signs of connective tissue and fatty replacement of the posterior muscles of the thighs and lower legs. Edema was noted in the anterior and medial muscle groups of the thighs, lower legs, and the multifidus muscle of the back. Whole genome sequencing revealed previously described mutations in the *DYSF* gene in exon 39 (c.4282 C > T) and intron 51 (c.5785-824 C > T). An immunohistochemical analysis and Western blot showed the complete absence of dysferlin protein expression in the muscle fibers.

**Conclusions:**

This case expands the range of clinical and phenotypic correlations of dysferlinopathy and complements the diagnostic search for spine rigidity.

**Supplementary Information:**

The online version contains supplementary material available at 10.1186/s12891-024-07270-y.

## Background

Dysferlinopathy is a phenotypically heterogeneous group of hereditary myopathies caused by mutations in the *DYSF* gene that has been mapped to locus 2p13.3-13.1 [1]. Deficiency of dysferlin (a transmembrane protein) leads to impaired repair of the sarcolemma and impaired rhabdomyogenesis [2–4]. In dysferlinopathy, symptoms of skeletal muscle damage have been described in relation to their main phenotypes [5]. While there has been little attention paid to the features and prevalence of articular contractures, it is believed that joint contractures in dysferlinopathy are uncommon [6] and occur in 11.9% [7] to 36% of cases [8]. However, the development of contractures of the ankle joints with the involvement of the Achilles tendon, hip, knee and elbow joints, fingers, and spine is possible [5, 7–9]. Articular contractures may develop as diffuse muscle damage progresses [10] and occur following outpatient status [7]. Early contractures are considered rare [11, 12], and rigid spine syndrome (RSS) in dysferlinopathy has been previously reported only once [13].

RSS was first proposed by Dubowitz [14, 15] to describe a subgroup of patients suffering from congenital myopathy with characteristic signs, i.e., early contracture of the spine and respiratory failure. Subsequently, this form was described as a congenital muscular dystrophy with early spinal rigidity (RSMD1) caused by a mutation in the *SEPN1* gene [16, 17] and a nosology similar to neurosis—multisystemic selenoprotein deficiency (*SECISBP2*) [18]. Spinal rigidity is a nonspecific sign in other myopathies caused by mutations in the following genes: *LMNA* [19, 20], *COL6* [21], *АСТА1* [22], *FHL1* [23], *MYH7* [19], *RYR1* [19], *LAMA2* [19], *CAPN3* [24], *TOR1AIP1* [25], *TK2* [26], *GAA* [19, 27], and *TPM3* [28].

In this study we present a case with an atypical phenotype of Miyoshi myopathy (MM) with spine rigidity and generalized articular contractures without respiratory insufficiency. The early manifestation of distal contractures of the upper and lower extremities coincided with the onset of rapidly progressive atrophy of the calf muscles.

## Case presentation

### Patient examination

The patient was a 23-year-old male, born from a 3rd pregnancy at 39 weeks, without perinatal pathology, from clinically healthy Russian parents. The second sibling (a 27-year-old female) was clinically healthy. A family history uncovered no neuromuscular or diffuse connective tissue diseases.

The patient exhibited a normosthenic body type, with a body mass index of 22.15. He began walking at the age of 1.2 years, developed normally according to his age, and was engaged in weightlifting until the age of 18. From the age of 13, he noted progressively increasing symmetrical contractures of the flexors of the fingers and elbow flexors of the wrists, as well as increasing weakness in the calf muscles. At the age of 14, a sharp atrophy of the calf muscles developed, which led to contractures of the Achilles tendons and limitations when walking on his heels. During this period, the patient noted general weakness, sweating, and shortness of breath during daily physical activity. From the age of 18, he noted a slowdown in running speed, and by the age of 20, he experienced fatigue of the thigh muscles when climbing stairs and standing from a sitting position. At the age of 22, the patient received glucocorticosteroids (prednisolone, 60 mg tablets q.d.) for one month, which led to a slight reduction in thigh muscle strength that was recovered after the medication was stopped. At the time of his examination, the patient was unable to rise on his toes, and contractures of the long extensors of the toes had formed, the most pronounced occurring on the right extensor halluces longus. At the age of 23, weakness appeared in the muscles of the shoulder girdle and the flexors of the fingers. A neurological examination revealed myogenic, predominantly distal tetraparesis. A moderate decrease in muscle strength (a 4/5 on the Medical Research Council (MRC) scale for muscle strength) was found in the deltoid muscles, deep finger flexors, thigh flexors and adductors, leg extensors, and ankle flexors. A more pronounced paresis was observed in the extensors of the feet (3/5 MRC). Carpal dynamometry was 21/15 kgf.

**Fig. 1:**
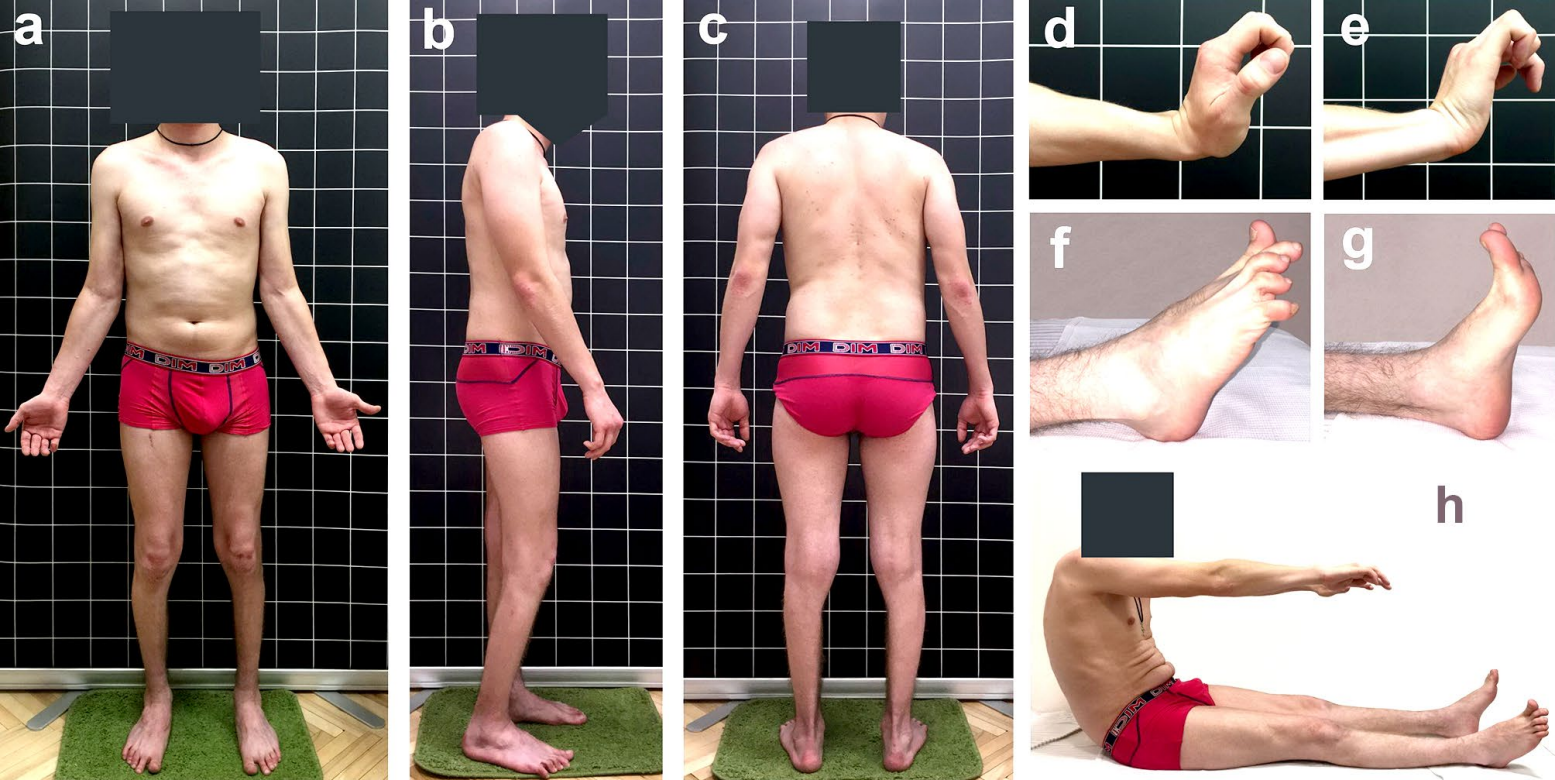
The atypical phenotype of a 23-year-old male with Miyoshi myopathy (**a**, **b**, **c**); flexion contractures of the fingers (**d**, **e**); extensor contractures and flexion of the toes (**f**); Achilles tendon contractures during foot extension (**g**); spine stiffness (**h**)

The most pronounced amyotrophies were observed in the posterior muscle group of the legs, with moderate amyotrophies in the posterior muscle group of the thighs. Elbow flexion, as well as Achilles and plantar reflexes, were absent. The most pronounced contractures were noted in the flexors of the fingers (up to 45° of the flexion position in the metacarpophalangeal joints with extended wrist joints), moderate contractures of the ulnar flexors of the wrists (up to 20°), and extensors of the fingers (up to 75° of the flexion position). Recurvation up to 5–7° was noted in the elbow joints. In the distal area of the lower extremities, pronounced contractures were detected in the ankle joints due to retraction of the Achilles tendons (flexion 110°, extension 100/98°) and the toe extensors, which formed a ‘hammer-like’ deformity of the digits. Moderate extensor contractures were noted in the hip joints (up to 100° flexion) due to the retraction of the semimembranosus muscle. Pronounced flexion contractures were also detected in the knee joints up to 110/120°. Rigidity of the spine was noted in the thoracic and lumbar regions and was accompanied by a limitation of trunk flexion to 60–70° (Fig. 1).

## Laboratory and imaging analyses

At the initial examination, the patient was 22 years old and presented with elevated levels of serum creatine kinase (CK) (44,060 U/l; normal value = 174 U/l), alanine aminotransferase (ALT) (209.3 U/l; normal range = 0.0–55.0 U/l), and aspartate aminotransferase (AST) (161.9 U/l; normal range = 5.0–34.0 U/l) during active weightlifting training. Following the cessation of training, the level of CK decreased to 19,000 U/l, ALT to 376.4 U/l, AST to 204.5 U/l, and lactic dehydrogenase (LDH) was 761.6 U/l (normal range = 120.0–246.0 U/l). While taking glucocorticosteroids at 22 years of age, the level of CK increased to 29,102 U/l. At the time of the current examination (when the patient was 23 years old), the CK level was 33,463 U/l, CK-MB (myocardial band) was 816.5 U/l, ALT was 510.5 U/l, AST was 431.2 U/l, LDH was 1199.2 U/l, and myoglobin was 4392.0 ng/ml (normal range = 0.0–0.8 ng/ml). All autoimmune markers (e.g., ANA (antinuclear antibodies)/ENA (extractable nuclear antigen), rheumatoid factor, total immunoglobulins, immunoblot of myositis-associated antibodies, and HLA-B27) were negative.

An electromyography (EMG) did not reveal impaired nerve conduction velocity but did show myopathic changes. An electrocardiography (ECG) uncovered concentric remodeling of the left ventricle, although the heart chambers were not enlarged. The patient’s vital capacity (5.1 L) and airway patency were within normal limits.

Magnetic resonance imaging (MRI) was performed using a Philips Ingenia 1.5 T MR scanner (Philips Medical Systems, Crawley, UK) using a surface coil. The protocol included T1, T2, and Short Tau Inversion Recovery (STIR) pulse sequences in three standard mutually perpendicular planes, with 30 slices at a thickness of 7 mm. Detected changes were assessed according to the scale by Mercuri [29].

**Fig. 2:**
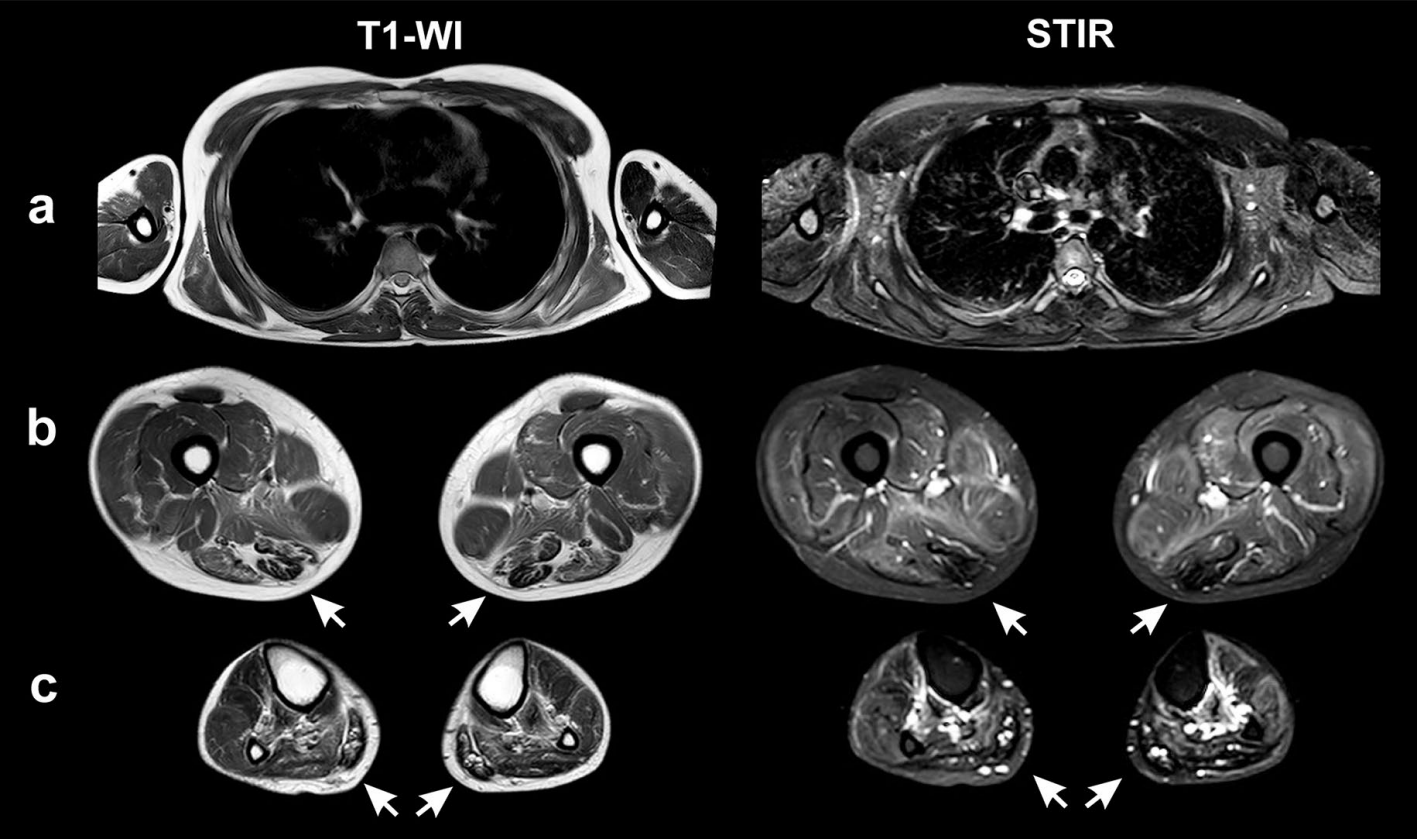
Magnetic resonance imaging (T1-WI, STIR) of the muscles of the trunk and shoulders (**a**), thighs (**b**), and lower legs (**c**). The arrows indicate muscles undergoing fibrosis

A whole-body MRI showed symmetrical and diffuse increases in the MR signal on T2-weighted images (T2-WI) and STIR in all muscle groups of the pelvic girdle and thighs due to moderate edematous changes in the muscles of the anterior, posterior, and medial muscle groups of the thighs. In addition, there were areas of linear and diffuse connective tissue (hypointense on STIR and T1-WI) and, to a lesser degree, fat replacement (hyperintense on T1, T2-WI, and hypointense on STIR). More pronounced areas of connective tissue and fatty degeneration were found in the posterior thigh muscle group (semimembranosus, semitendinosus, biceps femoris cup. longus; the 2b–3 stage by Mercuri). Less pronounced areas of fatty degeneration were noted in the anterior muscle group (vastus lateralis, vastus medialis, vastus intermedius; the 2a stage). The gracilis, sartorius, rectus femoris, and biceps femoris cup. brevis were characterized by moderate edematous changes, while the gracilis and sartorius muscles were also hypertrophied (8.0 cm^2^ and 5.4 cm^2^, respectively). Among the muscles of the legs, pronounced fibrous replacement was noted in the soleus and gastrocnemius muscles (2b–3 stage). In contrast, less pronounced changes were found in the tibialis posterior and the muscles of the anterior group (tibialis anterior, extensor digitorum, and peroneus longus; the 2a stage). Moderate edematous changes were observed in the tibialis posterior and anterior muscles on both the right and left sides. In the muscles of the shoulder girdle (supraspinatus, infraspinatus, and subscapularis) and in the muscles of the shoulder, moderate edematous changes were also detected. Moreover, the supraspinatus, infraspinatus, subscapularis, and teres major exhibited areas of fatty degeneration on both sides (stages 1–2a) (Fig. 2).

### Ethical procedure for the clinical examination

A 23-year-old male from a nonconsanguineous Russian family presenting with distal myopathy was examined. Clinical and neurological examinations and genealogical analyses of the patient’s family were carried out. All analyses were conducted after the patient signed a voluntary, informed consent form. Examinations were performed in accordance with relevant guidelines and regulations stated in the Declaration of Helsinki.

### Pathological findings

A biopsy specimen was obtained from a moderately affected right vastus lateralis muscle from an area of pronounced edematous changes according to the MRI scan (STIR). Frozen sections of the specimen were stained with hematoxylin and eosin, trichrome (according to Gomori), Sudan III, nicotinamide adenine dinucleotide (NADH), vytochrome c oxidase (COX), and superoxide dismutase (SDH), as well as immunohistochemically with antibodies to dysferlin (ab124684, Abcam, UK), CD45, CD68, HLA-ABC, and HLA-DR (Abcam, UK). A biopsy specimen of the vastus lateralis muscle from a healthy 26-year-old male was obtained as a control. One biopsy section was processed for transmission electron microscopy using the standard method [30].

The right vastus lateralis muscle biopsy revealed a myopathic pattern of changes as groups of rounded atrophic fibers, necrotic fibers, fibers with a central arrangement of nuclei, and pronounced endomysial and perimysial fibrosis. Furthermore, large droplet intermuscular lipoidosis was determined using Sudan III. Gomori’s trichrome uncovered single torn red fibers and atrophic fibers with increased numbers of mitochondria. Single COX-negative fibers were also detected via COX + SDH staining. Staining for NADH revealed an irregular internal architecture of muscle fibers.

**Fig. 3:**
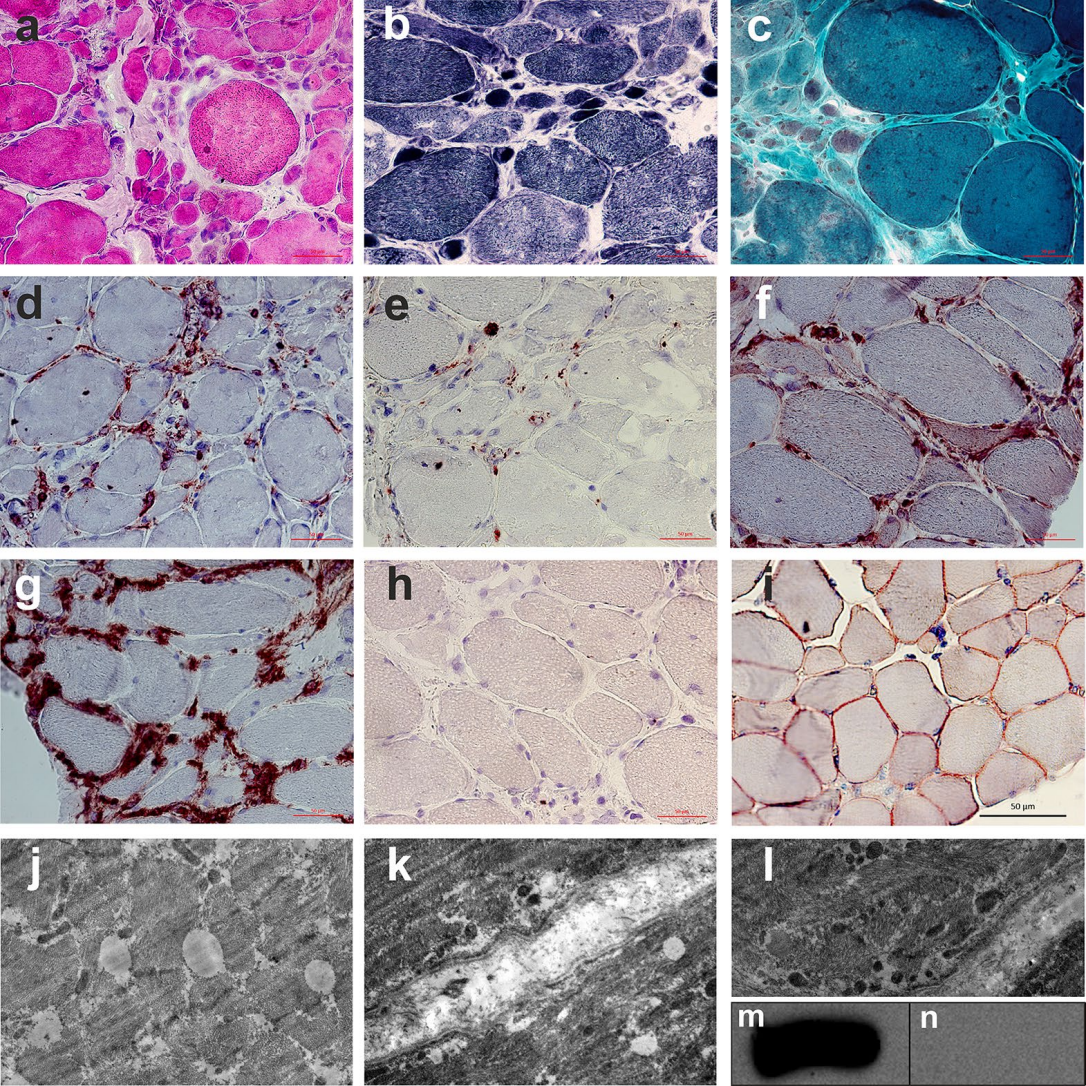
Pathological examination of the right vastus lateralis muscle biopsy specimen. Hematoxylin and eosin staining (**a**); NADH (**b**); trichrome Gomori (**c**); anti-CD45 (**d**); anti-CD68 (**e**); anti-HLA-ABC (**f**); anti-HLA-DR (**g**); anti-dysf (**h**); control (healthy donor) anti-dysf (**i**); ultrastructural signs of large droplet dystrophy with lipid inclusions (**j**); focal destruction of the sarcolemma (**k**); mitochondria are small and electron-dense (**l**); western blotting shows a complete absence of the dysferlin protein (**n**) when compared to the control (**m**)

​ An immunohistochemical analysis of the composition of the lymphocytic-macrophage infiltrate confirmed the presence of a pronounced endomysial cell infiltrate, indicating severe inflammation predominantly represented by lymphocytes. A partial violation of the integrity of the sarcolemma was also found, which consisted of an expansion of the subsarcolemmal spaces with flocculent detritus. The basement membrane of the muscle fiber was unevenly expanded and had formed thickenings, disturbing the homogeneity of the membrane. The mitochondria of the muscle fibers were also subject to changes, where subsarcolemmal and interfibrillar accumulations of polymorphic, small mitochondria with an electron-dense matrix were observed (Fig. 3).

### Western blot analysis

Polyacrylamide gel electrophoresis and Western blotting (WB) were performed as described previously [30]. All tissue samples were weighed, frozen, and homogenized in 19-volume electrophoresis buffer (e.g., 20 mg + 380 µl buffer) to give a loading concentration of approximately 2 mg in 30 µl [31]. The WB and immunohistochemical reaction with antibodies to dysferlin showed a complete absence of dysferlin expression in the muscle fibers (Fig. 3).

### Molecular genetic findings

The patient’s DNA sample was analyzed using whole genome sequencing (WGS) (next-generation sequencing). The mean read coverage for whole exome sequencing in all samples was 78.7×, with a read length of 2 × 75 bp. WGS had an average read coverage of 44.9×, with a read length of 2 × 151 bp. To estimate population frequencies of the identified variants, samples from the 1000 Genomes project, ESP6500, and the Genome Aggregation Database were used. The PolyPhen, SIFT (Sorting Intolerant From Tolerant), and MutationTaster programs were used to predict the possible effect of amino acid substitutions and protein functions. The number of copies of the 40 exons of the *DYSF* gene was examined using the multiplex ligation-dependent probe amplification (MLPA) method (The SALSA MLPA Probemix P268-A3 *DYSF* assay; MRC Holland, The Netherlands). Annotation of the exons was carried out according to the assembly LRG_845 (https://www.lrg-sequence.org). Potentially pathogenic mutations were identified via comparisons with the normal human genome, and detected changes were confirmed by Sanger sequencing.

A previously described, pathogenic mutation in the heterozygous state in the 39th exon of the *DYSF* gene (chr2:71839831 C > T, rs769721856; c.4282 C > T) was detected, leading to the appearance of a premature translation termination site in codon 1428 (p.Gln1428Ter, NM_001130987.1). The frequency of the detected mutation in the control sample of gnomAD was 0.0014%. WGS also revealed a previously described heterozygous mutation in the *DYSF* gene, which led to a change in the nucleotide sequence in intron 51 (chr2:71900503 C > T, c.5785-824 C > T, NM_001130987.1). The mutation was not registered in the control samples: 1000 genomes, ESP6500, ExAC, or gnomAD. According to the SpliceAI prediction algorithm, a mutation could result in an alternative splice acceptor site (SpliceAI_DS_AG: 0.51) or an alternative splice donor site (SpliceAI_DS_DG: 0.67). Therefore, based on the totality of the evidence, the mutation should be regarded as a variant of uncertain clinical significance. The patient had two copies of all exons of the *DYSF* gene by MLPA, which indicated the absence of large deletions. The patient’s mother was found to be a carrier of the c.4282 C > T mutation, and the father had another mutation (c.5785-824 C > T) in the heterozygous state.

## Discussion and conclusions

Our 23-year-old patient with MM and associated with multiple contractures and spine rigidity without respiratory insufficiency was clinically similar to a previously described case by Nagashima et al. [13] of the proximal-distal phenotype, which reinforces the possibility of a new variant of dysferlinopathy. However, a number of differences exist between our patient and the one reported by Nagashima et al. For instance, in our case, the manifestation of distal contractures was accompanied by the simultaneous development of muscular-dystrophic syndrome in the calf muscles from the age of 13. It is possible that weightlifting accelerated the progression and severity of contractures and muscle weakness [32]. Whereas Nagashima et al. [13] presented a case with a late onset of muscular dystrophic syndrome (a 40-year-old patient), with a primary manifestation of rigidity in the thoracic spine and an increasing development of multiple articular contractures. In our case, rigidity in the cervical region was not detected, possibly due to the relatively early stage of the disease. Furthermore, the CK level was significantly higher (253 times the upper normal limit) than in the Nagashima et al. case (25 times the upper normal limit). The use of glucocorticosteroids at the age of 22 led to slightly reduced muscle strength (which was reversible) and increased CK levels, as previously described in dysferlinopathy patients with a false diagnosis of polymyositis [33–35].

The patterns of fatty distribution and connective tissue infiltration of the muscles (observed via MRI and computed tomography) also differed from the case presented by Nagashima et al. In our patient, pronounced fibrotic changes were noted in both atrophied heads of the gastrocnemius and soleus muscles. In the Nagashima et al. case, minimal fat replacement and relative hypertrophy of the lateral portion of the gastrocnemius muscle were noted. Fatty and connective tissue infiltration of the thighs in both cases corresponded to a posterior-dominant distribution [36, 37], with the key aspect being the symmetrical, fibrosing nature of the change in the semitendinosus, which limits hip flexion. In our patient, there were no signs of fatty replacement of the paravertebral muscles, which is in contrast to the Nagashima et al. patient, who exhibited pronounced fatty replacement at the lumbar and thoracic levels [13]. Thus, spine rigidity in both cases indicates a fibrosing process that is likely independent of the degree of fatty replacement of the paravertebral muscles. Fanin et al. [5] have suggested that RSS, along with predominant lesions of the axial muscles, be included in the spectrum of dysferlinopathy symptoms. However, in none of the cases of axial muscle damage has pronounced tendon contractures and spine rigidity without respiratory failure been recorded [38, 39], even with total fatty replacement of the paravertebral muscles [39]. Pulmonary function was normal in our patient, in contrast to the Nagashima et al. patient, who exhibited an elevation in the right dome of the diaphragm [13].

The compound-heterozygous missense mutations in exons 39 and 51 caused a complete absence of dysferlin protein expression in the muscle fibers, which suggests the possibility of developing a pronounced fibrosing process in the absence of protein expression. However, the G3370T missense mutation in the dysferlin gene, also identified in the Nagashima et al. patient [13], is one of the most common in Japanese patients with MMs, among whom spine rigidity has not been found [40].

The variability in muscle fiber size, a small number of necrotic and torn red muscle fibers, and the presence of single COX-negative fibers are comparable to the reports by Nagashima et al. [13] and other researchers [41–43]. However, more pronounced endomysial and perimysial fibrosis were observed in our case. Furthermore, in contrast to macrophages being predominant in the composition of cellular infiltrates in dysferlinopathy [44–46], we uncovered pronounced lymphocytic and, to a lesser extent, macrophage (CD68+) infiltrates. Moreover, the endomysial and perimysial nature of the infiltrates did not differ from those previously described [33, 47–49]. Expression of major histocompatibility complex 1 (MHC1) on the surface of invaded and atrophied muscle fibers can be observed in 71.4% of cases of dysferlinopathy [44].

The complete absence of dysferlin in the skeletal muscles of our patient was associated with the presence of lipid droplets in myofibrils and small, electron-dense mitochondria, which corresponds to previously described alterations in lipid metabolism and oxidative processes in patients with dysferlinopathy and dysferlin-deficient mice [50, 51].

Multiple contractures, in combination with spine rigidity with or without respiratory failure, can be defined as a fibrosing phenotype that develops based on the distal distribution of muscular dystrophy, MM, and in the proximal-distal variant [13]. This clinical case expands the understanding of the phenotype and spectrum of clinical and genetic correlations characteristic of dysferlinopathy.

### Electronic supplementary material

Below is the link to the electronic supplementary material.

Supplementary Material 1

## Data Availability

The datasets analyzed during the current study are available in the ClinVar of the National Center for Biotechnology Information (Accession: VCV000285153.9; VCV000496981.11).

## Abbreviations

ALT: Alanine aminotransferase
ANA: Antinuclear antibodies
AST: Aspartate aminotransferase
CD: Cluster of differentiation
CK: Creatine kinase
COX: Cytochrome c oxidase
ECG: Electrocardiography
EMG: Electromyography
ENA: Extractable nuclear antigen antibodies
LDH: Lactic dehydrogenase
MHC1: Major histocompatibility complex 1
MLPA: Multiplex ligation-dependent probe amplification
MRC: Medical research council
MRI: Magnetic resonance imaging
NADH: Nicotinamide adenine dinucleotide
RSMD1: Muscular dystrophy with early spinal rigidity
RSS: Rigid spine syndrome
SDH: Superoxide dismutase
SIFT: Sorting intolerant from tolerant
STIR: Short tau inversion recovery
T2-WI: T2-weighted images
WB: Western blotting
WGS: Whole genome sequencing
ММ: Miyoshi myopathy
