# Supplementary figures and images for: Miyoshi myopathy associated with spine rigidity and multiple contractures: a case report
Source: BMC Musculoskelet Disord. 2024 Feb 16;25:146. doi: 10.1186/s12891-024-07270-y (PMC10870593; doi:10.1186/s12891-024-07270-y)

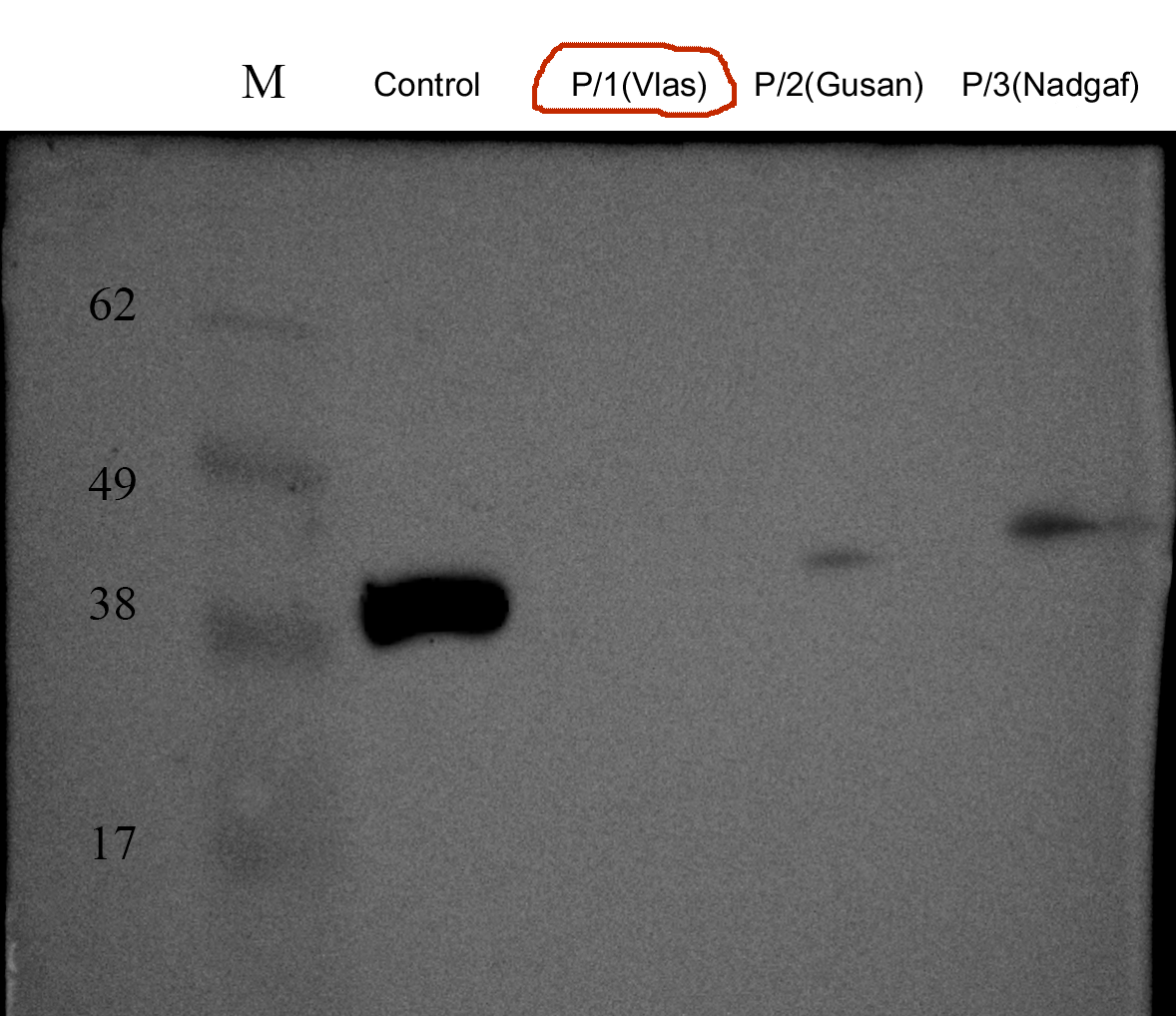

Supplement: Supplementary file 1 — Supplementary Material 1 [file 12891_2024_7270_MOESM1_ESM.tif]
